# Características Clínicas y Pronóstico Funcional en Pacientes con Encefalitis Autoinmune Posible en un Servicio de Urgencias Neurológicas

**DOI:** 10.31083/RN36202

**Published:** 2025-03-05

**Authors:** Paula Catalina Robles-Monroy, Victoria Martínez-Angeles, Jesús Ramírez-Bermúdez, Arturo Violante-Villanueva, Lilia Salas-Alvarado, Xiomara García, Juan Carlos López-Hernández

**Affiliations:** ^1^Departamento de Neuropsiquiatría, Subdirección de Psiquiatría, Instituto Nacional de Neurología y Neurocirugía Manuel Velasco Suarez, 14269 Ciudad de México, Mexico; ^2^Departamento de Urgencias Neurología, Instituto Nacional de Neurología y Neurocirugía Manuel Velasco Suarez, 14269 Ciudad de México, Mexico

**Keywords:** encefalitis mediada por anticuerpos, servicio de urgencias, neuropsiquiatría, pronóstico, antibody-mediated encephalitis, emergency department, neuropsychiatry, prognosis

## Abstract

**Introducción:**

La encefalitis autoinmune representa una enfermedad neuropsiquiátrica grave que requiere un diagnóstico temprano. Este trabajo describe la frecuencia, características clínicas y paraclínicas en pacientes con encefalitis autoinmune posible (EAP) atendidos en un servicio de urgencias neurológicas, así como factores asociados a pobre pronóstico funcional al egreso.

**Material y Métodos:**

Estudio observacional tipo cohorte ambispectiva de pacientes con diagnóstico de EAP atendidos en un servicio de urgencias neurológicas en 2022. Se evaluaron características clínicas, paraclínicas, y desenlace funcional al egreso hospitalario a través de la escala de Rankin modificada (mRS): ≤2 puntos fue considerado como buen pronóstico). En el análisis estadístico utilizamos prueba de chi cuadrada, exacta de Fisher, *T* de Student y U de Mann-Whitney.

**Resultados:**

De 9046 pacientes, 31 (0.3%) cumplieron criterios de EAP. La edad promedio fue 28.4 ± 12.1 años, y el 51.6% fueron mujeres. Se observaron alteraciones cognitivas (90.3%), psicosis (74.2%), movimientos anormales (71%), catatonia (67.7%), crisis/estado epiléptico (64.5%, 19.4%) y disautonomías (58.1%). El 58.1% presento buen pronóstico funcional al egreso. Los factores asociados con mal pronóstico fueron: edad (24.8 ± 5.0 vs. 33.4 ± 16.8, *p* = 0.049), estado epiléptico (0% vs. 46.2%, *p* = 0.002) y cefalea (61.1% vs. 15.4%, *p* = 0.025).

**Conclusiones:**

La EAP representa un diagnóstico poco frecuente en un centro de urgencias neurológicas; edad, estado epiléptico y cefalea fueron asociados a pobre pronostico funcional al egreso.

## Abstract (en)

**Introduction:**

Autoimmune encephalitis (AE) represents a severe neuropsychiatric disorder that requires early diagnosis. This study describes the frequency, clinical characteristics, and paraclinical findings in patients with possible autoimmune encephalitis treated in a neurological emergency department, as well as factors associated with poor functional prognosis at discharge.

**Material and Methods:**

This was an observational ambispective cohort study including patients diagnosed with AE who were treated in a neurological emergency department in 2022. Demographic, clinical, and paraclinical characteristics were evaluated, along with functional outcomes using the modified Rankin scale (mRS); ≤2 points, was considered as good prognosis. In the statistical analysis we used the chi-squared test, Fisher's exact test, Student's *T*-test, and Mann-Whitney U test.

**Results:**

Out of 9046 patients, 31 (0.3%) met the criteria for probable autoimmune encephalitis (PAE). The average age was 28.4 ± 12.1 years and 51.6% were female. Cognitive alterations (90.3%), psychosis (74.2%), abnormal movements (71%), catatonia (67.7%), seizures/status epilepticus (64.5%, 19.4%), and dysautonomia (58.1%) were observed; 58.1% had a good functional prognosis. Factors associated with poor prognosis included older age (24.8 ± 5.0 vs. 33.4 ± 16.8, *p* = 0.049), status epilepticus (0% vs. 46.2%, *p* = 0.002), and lower frequency of headache (61.1% vs. 15.4%, *p* = 0.025).

**Conclusions:**

AE represents a rare diagnosis even in a neurological emergency center; older age, status epilepticus and absence of headache were associated with poor functional prognosis at discharge.

## 1. Introducción

La encefalitis es una enfermedad neurológica grave que se presenta usualmente como una encefalopatía de progresión rápida, acompañada de una gran variedad de síntomas neuropsiquiátricos [1]. Las causas infecciosas son las más reconocibles, sin embargo, los avances en la investigación sobre la encefalitis autoinmune en los últimos 10 años han identificado nuevos síndromes y biomarcadores que han transformado el enfoque de esta patología [2, 3, 4].

En 2016, Graus y colaboradores [1] desarrollaron un algoritmo diagnóstico basado en la evaluación clínica y estudios paraclínicos convencionales para el diagnóstico de encefalitis autoinmune posible (EAP), con el objetivo de evitar el retraso en el diagnóstico y tratamiento, debido a la dificultad de realizar y obtener resultados de pruebas inmunológicas en un corto periodo de tiempo. Los criterios diagnósticos para EAP representan el punto de entrada para dicho algoritmo, debiendo presentarse la sintomatología en un periodo considerado como subagudo (progresión rápida de menos de 3 meses) de alteraciones en la memoria de trabajo, estado mental alterado y/o síntomas psiquiátricos, y al menos uno de los siguientes hallazgos: nuevos datos de focalización neurológica, crisis epilépticas no explicadas por un diagnóstico previo de epilepsia, pleocitosis en líquido cefalorraquídeo (LCR), hallazgos en resonancia magnética sugestivos de encefalitis y la exclusión razonable de otra causa [1].

La EAP es relevante debido a que los pacientes presentan un amplio espectro de síntomas que varían de intensidad, curso y pronóstico según la etiología. Por ejemplo, en la encefalitis anti receptor de N-metil-D-aspartato (NMDAR), la gran mayoría de los pacientes tienen síntomas psiquiátricos y/o conductuales prominentes que pueden ser difíciles de diferenciar de un trastorno psiquiátrico primario; el 80% de estos pacientes mejoran con inmunoterapia, aunque la recuperación es paulatina [3]. La encefalitis autoinmune se ha asociado con diferentes anticuerpos contra antígenos de superficie celular: NMDAR, Leucine-rich glioma inactivated 1 (LGI1), α-amino-3-hydroxy-5-methyl-4-isoxazolepropionic acid receptor (AMPAR), Contactina ssociated protein–like 2 (CASPR2), Gama-amino butyric acid receptor, type B (GABA_B_R) así como anticuerpos contra proteínas intracelulares Hu, Ma2 y glutamic acid decarboxylase 65 (GAD65); la diferenciación de estos dos grupos es relevante, puesto a que estos últimos responden pobremente a tratamiento inmunomodulador [5].

El objetivo del estudio es describir la frecuencia de pacientes con EAP, sus características clínicas y paraclínicas al ingreso y durante la estancia hospitalaria en un servicio de urgencias neurológicas, así como identificar factores asociados a pobre pronóstico funcional al egreso.

## 2. Material y Métodos

Estudio observacional tipo cohorte ambispectiva de pacientes atendidos en el Departamento de Urgencias Neurológicas en un centro de tercer nivel, durante el periodo de tiempo 01 de enero al 31 de diciembre del 2022. Fueron incluidos pacientes ≥18 años, que ingresaron con diagnóstico de encefalitis y/o encefalopatía de presunta etiología infecciosa o autoinmune [4], y que durante su evaluación clínica y paraclínica en el departamento de urgencias cumplieron los criterios de EAP [1].

Información de variables clínicas fueron recabadas: edad, género, tiempo de inicio de los síntomas al ingreso (días), infección previa (definida como proceso infeccioso inespecífico de vías respiratorias o gastrointestinal hasta 30 días previos al inicio del cuadro de EAP [6, 7]), fiebre, síntomas o signos neurológicos (cefalea, encefalopatía, crisis epilépticas, estado epiléptico, alteraciones cognitivas, discinesias orolinguales), síntoma psicóticos, catatonia (por evaluación clínica y ≥2 puntos de screening en la escala de Bush y Francis [8]) y presencia de disautonomías cardiovasculares (definida como taquicardia, bradicardia, hipertensión o hipotensión arterial sostenida, no explicada por sepsis, fiebre o fármacos, a criterio de médico tratante).

De los estudios paraclínicos, fueron recabados los resultados de creatinin-fosfoquinasa (CPK) (en la actualidad es considerado como un biomarcador de interés en los procesos sistémicos y neurológicos de origen inflamatorio) [9, 10, 11], un aumento >223 U/L fue considerado anormal (valor de referencia normal <223 U/L). Además, fueron recabados los reportes de electroencefalograma (EEG) al ingreso (clasificándolos en: normal, disfunción o con actividad epiléptica) [12]; también se obtuvieron los resultados de estudios de LCR al ingreso, tanto de citoquímico (considerando, recuento >5 células por mm^3^ como pleocitosis y niveles de proteínas >45 mgs/dL como hiperproteinorraquia), así como el resultado de anticuerpo anti-NMDAR [1].

Los hallazgos de la resonancia magnética de encéfalo y resultado de bandas oligoclonales en LCR no fueron incluidos en el estudio, dado a que su realización y/o reporte de resultado presentan un retraso de 1–2 semanas en nuestra institución. Debido a las limitaciones de nuestro centro, solo fue realizado y recabado el estudio anticuerpos anti-NMDAR en LCR, por lo que los pacientes fueron clasificados en encefalitis anti-NMDAR definida (criterios de Graus y colaboradores [1]) o EAP con anticuerpos anti-NMDAR negativo.

**Fig. 1.**
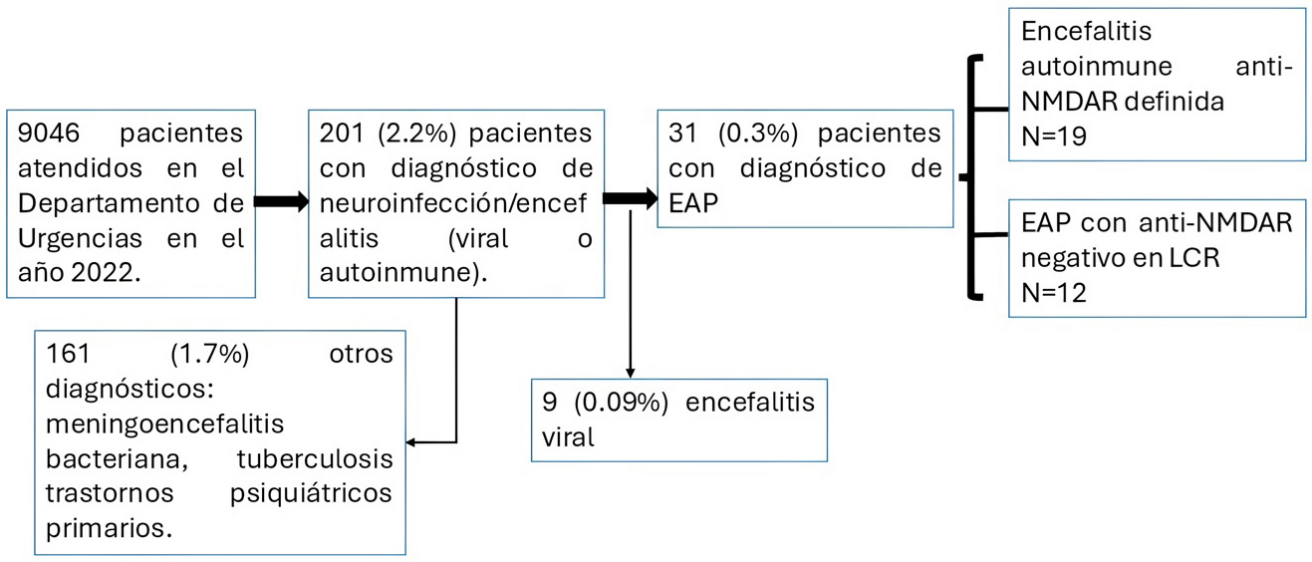
**Pacientes incluidos y excluidos en el estudio**. EAP, encefalitis autoinmune posible; LCR, líquido cefalorraquídeo; anti-NMDAR, anticuerpo-anti receptorde N-metil-D-aspartato.

Los pacientes fueron seguidos hasta su egreso hospitalario para registrar: diagnóstico final y puntuación en la escala de Rankin modificada (mRS) al egreso, tratamiento inmunomodulador recibido (bolos de metilprednisolona y/o recambios plasmáticos), resultados de anticuerpos para enfermedades autoinmunes sistémicas (anticuerpos antinucleares por inmunoflorencia indirecta (IFI), anti-DNA doble cadena, anticardiolipinas inmunoglobulina G (IgG) e inmunoglobulina M (IgM), anti beta2 glicoproteína IgG e IgM, anticoagulante lúpico, anti-SSA y anti-SSB), hallazgo de tumoración asociada y días de estancia intrahospitalaria (DEIH). Fueron excluidos del análisis final, aquellos pacientes cuya evolución y resultado de estudios paraclínicos posteriores (imagen de resonancia magnética de encéfalo, panel FilmArray para patógenos causantes de meningoencefalitis, cultivos de LCR, estudio de GeneXpert en LCR, etc.) cumplieron diagnóstico para: encefalitis viral, meningoencefalitis bacteriana o asociada a infección por virus de la inmunodeficiencia humana (VIH), tuberculosis meníngea, trastorno psiquiátrico primario, etc. (Fig. 1).

Los pacientes que presentaron al egreso hospitalario una puntuación en la escala mRS ≥3 puntos fueron considerados con pobre pronostico funcional [13].

### Análisis Estadístico

Para el análisis descriptivo se determinó la distribución de las variables continuas con la prueba de Kolmogorov-Smirnov, describiéndose en promedios con desviación estándar (DE) o medianas con rango intercuartílico (RIQ), según su distribución. Las variables categóricas se describieron en frecuencias y porcentajes. Para analizar diferencias entre grupos se utilizó: prueba *x*^2^ y exacta de Fisher para variables categóricas, prueba *t* de Student para comparar promedios y para comparar medianas prueba U de Mann-Whitney. Un valor de *p* < 0.05 fue conciderado estadísticamente significativo. Todos los análisis fueron realizados en el programa estadístico SPSS versión 22.0 (IBM Corporation, Armonk, NY, USA).

## 3. Resultados

Fueron atendidos 9046 pacientes en el Departamento de Urgencias durante el año 2022, 201 (2.2%) ingresaron con diagnóstico de encefalitis y/o encefalopatía de presunta etiología infecciosa o autoinmune, al egreso hospitalario 31 pacientes (0.3%) cumplieron criterios diagnósticos de EAP (19 pacientes con encefalitis anti-NMDAR definida, 12 pacientes con EAP con anticuerpos anti-NMDAR negativo) (Fig. 1). Las principales características de los pacientes con EAP fueron: edad de 28.4 ± 12.1 años, sexo femenino 51.6%, inicio de los síntomas al ingreso (mediana) 15 (RIQ 5–39) días, DEIH (mediana) 45 (RIQ 22–65) días, antecedente de infección previa 35.5%, alteraciones cognitivas 90.3%, síntomas psicóticos 74.2% y estado epiléptico 19.4%. El resto de las características clínicas y hallazgos paraclínicos se presentan en la Tabla 1. Dos pacientes (6.5%) presentaron hallazgo de tumoración, y durante el seguimiento fueron clasificados en teratoma de ovario maduro y nódulo pulmonar solitario. Los resultados de anticuerpos para enfermedades autoinmunes sistémicas fueron negativos en todos los pacientes.

**Tabla 1.** **Características clínicas, paraclínicas y pronóstico funcional de los pacientes con Encefalitis Autoinmune Posible**.

		N = 31
Edad (años), promedio (DE)		28.4 ± 12.1
Sexo femenino, n (%)		16 (51.6)
Días del inicio de los síntomas al ingreso, mediana (RIQ)		15 (5–39)
Días de estancia hospitalaria, mediana (RIQ)		45 (22–65)
Características clínicas:		
	Infección previa, n (%)	11 (35.5)
	Cefalea, n (%)	13 (41.9)
	Fiebre, n (%)	14 (41.9)
	Síntomas psicóticos, n (%)	23 (74.2)
	Alteraciones cognitivas, n (%)	28 (90.3)
	Crisis epilépticas, n (%)	20 (64.5)
	Estado epiléptico, n (%)	6 (19.4)
	Catatonia, n (%)	21 (67.7)
	Movimientos anormales, n (%)	22 (71)
	Disautonomías, n (%)	18 (58.1)
	Diagnóstico de tumoración, n (%)	2 (6.5)
Características paraclínicas:		
	Células en LCR, mediana (RIQ)	6 (2–18)
	Pleocitosis, n (%)	17 (54.8)
	Proteínas (mg/dL) en LCR, mediana (RIQ)	24 (17–32)
	Hiperproteinorraquia, n (%)	8 (25.8)
	CPK (U/L), mediana (RIQ)	225.9/24 (103–471.2)
	CPK elevada, n (%)	11 (45.8)
EEG al ingreso:		
	Normal, n (%)	3/27 (11.1)
	Disfunción, n (%)	21/27 (77.8)
	Actividad epiléptica, n (%)	3/27 (11.1)
Tratamiento recibido:		
	Bolos de metilprednisolona, n (%)	29 (93.5)
	Recambios plasmáticos, n (%)	24 (77.4)
Pronostico funcional		
	mRS ≤2 puntos al egreso, n (%)	18 (58.1)
	Defunción, n (%)	2 (6.5)
Diagnostico al egreso hospitalario:		
	Encefalitis anti-NMDAR definida, n (%)	19 (61.2)
	EAP con anticuerpos anti-NMDAR negativos en LCR, n (%)	12 (38.7)

EAP, encefalitis autoinmune posible; DE, desviación estándar; RIQ, rango intercuartílico; LCR, líquido cefalorraquídeo; CPK, creatinin-fosfoquinasa; EEG, electroencefalograma; mRS, escala de Rankin modificada; NMDAR, anti receptor de N-metil-D-aspartato.

Con respecto al pronóstico funcional al egreso hospitalario, 18 pacientes (58%) presentaron buen pronóstico (mRS ≤2 puntos), y 13 pacientes (42%) presentaron pobre pronóstico (mRS ≥3 puntos), de los cuales 2 pacientes fallecieron (6.4%). Al comparar los grupos, observamos que la edad (24.8 ± 5.0 vs. 33.4 ± 16.8, *p* = 0.049), cefalea (61.1% vs. 15.4%, *p* = 0.025), estado epiléptico (0% vs. 46.2%, *p* = 0.002) y hallazgos en el EEG de ingreso (*p* = 0.046) presentaron diferencias estadísticamente significativas. El resto de las diferencias clínicas y paraclínicas entre pacientes con EAP con buen pronóstico y pobre pronóstico funcional se presentan en la Tabla 2.

**Tabla 2.** **Diferencias clínicas y paraclínicas entre pacientes con EAP con buen pronóstico y pobre pronóstico funcional**.

		mRS ≤2 puntos	mRS ≥3 puntos	Valor de *p*
		N = 18	N = 13	
Edad, promedio (DE)		24.8 ± 5.0	33.4 ± 16.8	0.049
Sexo femenino, n (%)		8 (44.4)	8 (61.5)	0.34
Días del inicio de los síntomas al ingreso, mediana (RIQ)		17 (3.7–32.5)	9 (5–40)	0.70
Días de estancia intrahospitalaria, promedio (DE)		40.8 ± 22.4	56.8 ± 30.4	0.10
Infección previa, n (%)		6 (35.3)	5 (38.4)	0.99
Cefalea, n (%)		11 (61.1)	2 (15.4)	0.02
Fiebre, n (%)		7 (38.9)	6 (46.2)	0.72
Síntomas psicóticos, n (%)		14 (77.8)	9 (69.2)	0.68
Catatonia, n (%)		13 (72.2)	8 (61.5)	0.70
Crisis epilépticas, n (%)		11 (61.1)	9 (69.2)	0.71
Estado epiléptico, n (%)		0 (0)	6 (46.2)	0.002
Alteraciones cognitivas, n (%)		16 (89.9)	12 (92.3)	0.99
Movimientos anormales, n (%)		11 (61.1)	11 (84.6)	0.23
Disautonomías, n (%)		11 (61.1)	7 (53.8)	0.72
Hallazgo de tumoración, n (%)		1 (5.6)	1 (7.7)	0.99
Células en LCR, mediana (RIQ)		9.5 (3.7–21.5)	6 (2–17)	0.59
Proteínas en LCR, mediana (RIQ)		22.5 (17.7–32.2)	27 (17.7–32.2)	0.54
CPK, mediana (RIQ)		301 (134.5–500.5)	129.5 (57.9–207.5)	0.08
Albúmina, promedio (DE)		4.5 ± 0.5	4.2 ± 0.6	0.16
EEG al ingreso:				
	Normal, n (%)	3 (20)	0 (0)	0.23
	Disfunción, n (%)	12 (80)	9 (75)	0.99
	Actividad epiléptica, n (%)	0 (0)	3 (25)	0.07
Tratamiento recibido:				
	Bolos de metilprednisolona, n (%)	16 (88.8)	13 (100)	0.49
	Recambios plasmáticos, n (%)	12 (66.7)	12 (92.3)	0.19
	Diagnóstico final de encefalitis anti-NMDAR definida, n (%)	14 (77.8)	5 (38.5)	0.06

## 4. Discusión

En nuestro servicio de urgencias neurológicas, la EAP es un diagnóstico poco frecuente (0.3%). Este hallazgo podría explicarse debido a que, siguiendo las recomendaciones de diferentes grupos de expertos, fueron considerados una amplia gama de diagnósticos diferenciales a descartar dentro del abordaje de los pacientes (meningoencefalitis bacteriana, tuberculosis del sistema nervioso central, trastornos psiquiátricos primarios, encefalitis virales, etc.), clasificando únicamente a los pacientes que cumplían criterios como EAP [2, 3, 13]. En nuestra población de pacientes con EAP, el diagnostico final más frecuente fue el de encefalitis anti-NMDAR definida, esto de acuerdo con la literatura [14, 15].

El tiempo (días) desde el inicio de los síntomas hasta el ingreso en urgencias fue de 15 días. En la práctica clínica es importante realizar un diagnóstico oportuno de esta patología, dado que, a menor número de días entre el inicio de los síntomas y el inicio del tratamiento inmunomodulador, es un factor de buen pronóstico [2]. La presentación clínica de los pacientes con EAP en nuestro centro fue similar a la reportada en la literatura [1, 2, 3, 16], con presencia de síntomas psiquiátricos prominentes, como son las alteraciones cognitivas (90.3%), síntomas psicóticos (74.2%) y catatonia (67.7%); además de síntomas neurológicos: presencia de movimientos anormales (71%), disautonomías (58.1%) y crisis/estado epiléptico (64.5% y 19.4% respectivamente).

En nuestro estudio, 58% de los pacientes con EAP presento un buen pronóstico funcional al egreso a través de la escala mRS, al igual que otros estudios [7, 17]. Sin embargo, es necesario considerar que, para la evaluación de estos pacientes durante su seguimiento, se requiere de la aplicación de otras escalas, dado a que los síntomas neuropsiquiátricos (trastornos del estado de ánimo, alteraciones del sueño, alteraciones de memoria, atención, función ejecutiva, etc.) son las secuelas más frecuentemente reportadas. Por lo anterior, se sugiere utilizar la escala Clinical Assessment Scale in Autoimmune Encephalitis (CASE) y la escala anti-NMDAR-encephalitis one-year functional status (NEOS) en estos pacientes [13, 15].

Sobre los resultados paraclínicos, nuestra población presento en LCR pleocitosis 54.8% e hiperproteinorraquia 25.8%. Los hallazgos de pleocitosis son consistentes con los reportados en otros centros, aunque los resultados de hiperproteinorraquia difieren. En un estudio retrospectivo realizado en la Universidad de Viena, se analizaron las características de LCR de 33 pacientes con encefalitis autoinmune definida por diferentes anticuerpos de superficie e intracelulares, reportando pleocitosis en 45.5%, e hiperproteinorraquia en 60.6% [18, 19]. La pleocitosis es considerada dentro de los criterios diagnósticos de EAP. La hiperproteinorraquia se ha considerado un marcador inespecífico de inflamación que puede alterarse en diferentes patologías del sistema nervioso central y actualmente no se encuentra dentro de los criterios para EAP [1]. Es importante considerar que las alteraciones en el LCR podrían verse influenciados por el tipo de encefalitis autoinmune, el tiempo de inicio de la enfermedad a la realización del estudio, la edad, etc. y su normalidad no descarta la EAP, requiriendo la determinación de autoanticuerpos específicos por técnicas recomendadas para el diagnóstico definitivo.

Sobre los niveles de CPK, 45.8% de pacientes presento niveles aumentados, aunque no existió diferencia significativa entre pacientes con buen y pobre pronóstico funcional (*p* = 0.081). Consideramos que los niveles elevados de CPK son a consecuencia de la agitación secundaria a psicosis, así como a crisis epilépticas y catatonia, sin embargo, adquieren un valor importante cuando se presenta posterior a la administración de antipsicóticos en este grupo de pacientes, por el riesgo aumentado de síndrome neuroléptico maligno [20].

El hallazgo de actividad epiléptica en el EEG en nuestra población fue poco frecuente, pese a que clínicamente 64.5% de los pacientes presentaron crisis epilépticas y 19.4% estado epiléptico. Consideramos que esto es debido a que el diagnóstico y manejo de estas entidades es principalmente clínico, por lo que en la gran mayoría de los casos ya se había iniciado un manejo farmacológico previo a la obtención del estudio. La realización de este estudio en pacientes que presentan síntomas neuropsiquiátricos prominentes (ej. psicosis) de inicio subagudo es recomendable, pues su anormalidad se puede considerar una “bandera roja” para sospechar autoinmunidad [20]. El EEG puede presentar anormalidad por hallazgos inespecíficos en 80 a 90% de los casos [16], sin embargo, su normalidad no descarta el diagnóstico de EAP.

En nuestra población, los pacientes con pobre pronóstico funcional presentaron mayor edad, menor frecuencia de cefalea como síntoma prodrómico y mayor frecuencia de estado epiléptico. La mayor edad se ha asociado con peor pronóstico en pacientes mujeres con diagnóstico de encefalitis anti-NMDAR definida, no así en hombres, lo cual concuerda con nuestro hallazgo en cuanto edad, no así en sexo [21]. La presentación de cefalea de reciente inicio o una modificación en su patrón previo, se ha identificado como una bandera roja para la presentación de encefalitis autoinmune [22], siendo descrita dentro de un cuadro prodrómico pseudo viral. En una serie de 100 pacientes con encefalitis, 86% tuvo cefalea, fiebre o síntomas semejantes a los de una infección viral, con posterior progresión de los síntomas neuropsiquiátricos [2, 3, 15]. En nuestra población, la cefalea se presentó en el 41.9% de los casos, siendo un hallazgo interesante, porque los pacientes con pobre pronóstico presentan menor frecuencia de este síntoma.

La presentación de cefalea ha sido descrita en diferentes entidades neurológicas y sistémicas inmunomediadas, por ejemplo, dentro de las manifestaciones del síndrome antifosfolípidos (SAF), donde la cefalea tiene una prevalencia del 20% y pudiendo ser de intensidad incapacitante. Los pacientes con SAF cursan con una gran variedad de trastornos neuropsiquiátricos, similares a los pacientes con EAP, dentro de los cuales destacan demencia, disfunción cognitiva, psicosis y depresión [23]. Igualmente, es relevante señalar que muchas de estas manifestaciones también se han descrito en Lupus Eritematoso Sistémico [24], lo que representa la necesidad de continuar investigando sobre los mecanismos involucrados de autoinmunidad y su asociación con los síntomas neuropsiquiátricos en estos padecimientos.

La presencia de crisis epilépticas aumenta la sospecha clínica de padecimientos encefaliticos, sobre todo cuando se acompañan de síntomas neuropsiquiátricos y de evolución aguda/subaguda. En distintas series publicadas de EA, la presencia de crisis epilépticas es del 80% [3, 16]. En el caso de la encefalitis anti-NMDAR, un estudio reporto 109 pacientes, en el cual se evaluó la presencia y el tipo de crisis epilépticas; 80.7% presento crisis en la fase aguda, crisis única 19.3%, crisis repetidas 30.7%, estado epiléptico 25%, estado epiléptico refractario 14.8% y estado epiléptico super refractario 10.2%. En nuestra población, observamos un menor porcentaje de crisis epilépticas (64.5%) y un menor porcentaje de estado epiléptico (19.4%), sin embargo y acorde a la literatura, el estado epiléptico se asoció con pobre pronóstico funcional al egreso. Esto es relevante debido a que, durante el curso clínico de la encefalitis autoinmune, la presencia de tumor, estado epiléptico, estado de coma o el ingreso a una unidad de cuidados intensivos durante la fase aguda, son factores asociados en la persistencia de crisis, una vez pasada la fase aguda de la enfermedad lo cual puede impactar a largo plazo en la funcionalidad [25].

### Limitaciones del Estudio

El estudio presenta varias limitaciones, primeramente, el número reducido de pacientes incluidos. Otra limitación, es que debido a la falta de recursos económicos en nuestra institución no contamos con la determinación de paneles de anticuerpos de superficie cerebral para determinar la causa de encefalitis autoinmune en pacientes con anti-NMDAR negativo. Además, dado el objetivo del presente estudio, no se incluyó información de otras variables: hallazgos en la resonancia magnética de encéfalo, tiempo de inicio de tratamiento inmunomodulador, complicaciones intrahospitalarias, que pueden influir en el pronóstico funcional al egreso. Por último, no contamos con información sobre sí los pacientes recibieron esquemas de tratamiento de segunda línea para la encefalitis autoinmune como rituximab o ciclofosfamida.

## 5. Conclusiones

La encefalitis autoinmune es un diagnóstico poco común incluso en un centro de urgencias neurológicas; sin embargo, debe considerarse como parte de los diagnósticos diferenciales en pacientes con sospecha de neuroinfección. Esta enfermedad presenta una amplia gama de síntomas neuropsiquiátricos potencialmente mortales, por lo que es crucial identificar de manera precisa los síntomas que puedan orientar su diagnóstico diferencial y realizar los estudios paraclínicos pertinentes. Mayor edad, ausencia de cefalea como síntoma prodrómico y la presencia de estado epiléptico se asociaron a un pobre pronóstico funcional al egreso.

## Data Availability

Los datos ya se proporcionan en el artículo.

## Contribuciones de los Autores

PCRM: diseño de investigación, adquisición e interpretación de los datos, redacción y revisión del manuscrito; VMA: diseño de investigación, adquisición e interpretación de los datos, redacción y revisión del manuscrito, edicion; JRB: diseño de investigación, interpretacion de lo datos, redacción y revisión del manuscrito; AVV: diseño de investigación, interpretacion de los datos, redacción y revisión del manuscrito; LSA: diseño de investigación, adquisición de los datos, redacción del manuscrito; XG: diseño de investigación, adquisicion de los datos, redacción del manuscrito; JCLH: diseño de investigación, interpretación de los datos, revisión, edición. Todos los autores leyeron y aprobaron el manuscrito final. Todos los autores han participado lo suficiente en el trabajo y han acordado ser responsables de todos los aspectos del mismo.

## Aprobación Ética y Consentimiento Informado

Este manuscrito se adhiera a las directrices de la Declaración de Helsinki. El protocolo de investigación fue aprobado por los Comités de Ética e Investigación del Instituto Nacional de Neurología y Neurocirugía Manuel Velasco Suárez (Número de Aprobación Ética: 168/22), y todos los participantes proporcionaron consentimiento informado firmado.

## Agradecimientos

No aplicable.

## Financiación

Esta investigación no recibió financiación externa.
